# Sociodemographic barriers to continuous glucose monitoring among pregnancies with type 1 diabetes

**DOI:** 10.1002/pmf2.70135

**Published:** 2025-10-22

**Authors:** Frank B. Will Williams, Kali Juracek, John A. Morgan, James D. Toppin, Joseph R. Biggio, Shannon M. McCloskey

**Affiliations:** ^1^ Division of Maternal Fetal Medicine Ochsner Health New Orleans Louisiana USA; ^2^ Ochsner Clinical School University of Queensland Medical School Brisbane Australia

**Keywords:** CGM, diabetes, disparities, pregnancy

## Abstract

**Objective:**

Continuous glucose monitor (CGM) use is associated with improved glycemic management among pregnant patients with type 1 diabetes. Individual‐ and neighborhood‐level social determinants of health (SDHs) are associated with low CGM use and adverse pregnancy outcomes. We hypothesized that SDHs are associated with decreased CGM use in pregnancy among patients with type 1 diabetes.

**Methods:**

We evaluated a cohort of pregnancies receiving type 1 diabetes and delivery care at a single large health system from 2016 to 2023. Patients were evaluated by public payor status. The primary outcome was CGM use. Additional SDH characteristics evaluated included home Area Deprivation Index (ADI) percentile and rurality status. Regression analyses generating models predicting CGM use included age, baseline body mass index (BMI), diabetes duration, and delivery year.

**Results:**

Among 288 pregnancies with type 1 diabetes, 144 (50.0%) had public insurance. Public payor was associated with younger age and shorter diabetes duration, and those patients were more commonly nulliparous and Black. Controlling for baseline characteristics, CGM uptake was lower among the public insured (38.9% vs. 61.8%; adjusted odds ratio [aOR], 0.39; 95% confidence interval [CI], 0.23–0.66). Over time, uptake increased, though publicly insured patients lagged by two years. Evaluating SDH characteristics and CGM usage over time, public insurance (aOR, 0.47; 95% CI, 0.24–0.91) and ADI (aOR, 0.96; 95% CI, 0.95–0.98) were associated with CGM use; rurality was not.

**Conclusion:**

Public insurance and high neighborhood deprivation are risk factors for lower CGM uptake, potentially representing an indirect and targetable mechanism by which SDH impacts glycemic management for pregnancies with type 1 diabetes.

## Graphical abstract

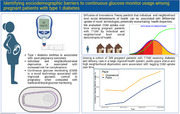

## 1 INTRODUCTION

Type 1 diabetes mellitus (T1DM) is a strong predictor of poor pregnancy outcomes, associated with congenital anomalies, preterm delivery, perinatal mortality, hypertensive disorders of pregnancy, and maternal mortality [1, 2, 3]. For those living with T1DM, pregnancy is associated with increased risk of diabetes‐related complications, including worsening nephropathy [4, 5] and retinopathy [6, 7] along with increased risk for diabetic ketoacidosis [8, 9]. Tight glycemic management in the context of increasing insulin resistance through gestation is central to achieving optimal pregnancy outcomes for patients with T1DM [10, 11, 12]. Even modest elevations above the target glycated hemoglobin (A1c) of 6.0% are associated with large for gestational age infants, preterm delivery, preeclampsia, and need for neonatal glucose infusion [10].

A number of social determinants of health (SDHs) have been associated with hyperglycemia, diabetic complications, and pregnancy complications for those with T1DM [13, 14]. Higher A1c values are observed in racial and ethnic minorities, those using public insurance, and those residing in less advantaged residential areas [15, 16, 17, 18, 19, 20]. While such disparities are multifactorial, a contributor to disparate improvements in outcomes among disadvantaged groups may be related to differences in uptake of technological innovations [21, 22]. Diffusion of Innovations theory postulates that new ideas, practices, and devices are adopted in a progressive manner across social groups, where those with higher social standing tend to adopt innovations earlier and accumulate a disproportionate advantage relative to those with lower social standing who adopt innovations later [23]. Thus, even when a new innovation improves health across a population, uneven adoption can exacerbate disparities [21]. Race, socioeconomic status, and rurality, each associated with social disadvantage in the United States, have been associated with lagging uptake of diabetic technologies in nonpregnant populations [15, 24, 25, 26], including continuous glucose monitoring (CGM) [26, 27].

Mounting evidence of improved glycemic management among people living with T1DM has led to increasing CGM uptake [16, 28]. From 2010 to 2019, commercially insured children and adults with T1DM increased their use of CGM from 20% to almost 50% [29]. The multicenter randomized controlled Continuous Glucose Monitoring in Pregnant Women with Type 1 Diabetes (CONCEPTT) trial has provided evidence of benefit in pregnancy, demonstrating that compared to standard care, addition of CGM resulted in improved A1c and reduced adverse neonatal outcomes and was cost‐effective [30, 31, 32]. Data collected before and during dissemination of CONCEPTT's findings demonstrated disparate use of CGM among reproductive‐age women by SDH, though at that time overall uptake was less than 30% [25]. In the wake of broader uptake of CGM, we hypothesized that individual‐ and neighborhood‐level markers of social disadvantage are associated with decreased CGM use among pregnant patients. We aim to evaluate the relationship between SDOH and CGM uptake among patients with T1DM receiving pregnancy care at a large regional healthcare system.

## 2 METHODS

This is a retrospective cohort study of all pregnant patients diagnosed with T1DM who received diabetes care and delivered within a large regional healthcare system in Louisiana between January 2016 and January 2023. Deliveries occurring in 12 hospitals in urban, suburban, and rural settings with both academic and community care models were included. The local Institutional Review Board approved the study, and design and reporting are in accordance with Strengthening the Reporting of Observational Studies in Epidemiology statement.

To identify patients carrying a diagnosis of type 1 diabetes, all recorded pregnancy episodes during the study period that had ever had an International Classification of Diseases (ICD); 10‐ Clinical Modification (CM) diagnosis for T1DM (E10.*) associated with a clinical encounter, either before pregnancy or during pregnancy, were queried from the electronic medical record (EMR). As incorrect EMR ICD‐10 documentation can be common (e.g., a patient with type 2 diabetes seen in an emergency department for a clavicular fracture or a patient who had gestational diabetes A1 at a postpartum visit), manual chart review of each patient was performed by one of two investigators (S.M.M., K.J.) to validate T1DM diagnosis, documented CGM status by 20 weeks gestation, and delivery outcome. Those establishing prenatal care after 20 weeks gestation, those delivering prior to 20 weeks, those with missing CGM status, and those lacking delivery outcomes were excluded. Those with CGM use documented in the EMR, either via provider description in a consult/progress note or via reports uploaded into the Media section of the EMR, before 20 weeks of pregnancy were classified as CGM users. Those not using CGM prior to 20 weeks, whether unprescribed or due to patient factors (including preference), were classified as nonusers. As above, individuals with T1DM whose documentation did not characterize method of glycemic monitoring prior to 20 weeks gestation were excluded from the study. Baseline and pregnancy characteristics collected included maternal age, nulliparity, body mass index (BMI) at presentation to pregnancy care, smoking status, chronic hypertension, duration of diabetes diagnosis, insulin pump use, and glycated hemoglobin at presentation to pregnancy care. Sociodemographic characteristics were evaluated at the individual and neighborhood levels. Individual low‐income status was defined by use of public insurance payor, which in our cohort primarily consisted of LaMOMS—Louisiana's publicly funded Medicaid insurer for pregnancy. Pregnant people with family income below 133% of the federal poverty level qualify for LaMOMS. Importantly, the payor has covered CGM for patients with insulin‐dependent diabetes since before the inclusion period, implying that payor CGM coverage would not be a primary mediator of differences in CGM uptake by insurance status. Neighborhood‐level socioeconomic disparities evaluated were based on home address associated with the pregnancy episode and included Area Deprivation Index (ADI) and rurality. The former is a measure of neighborhood deprivation measured at the census block level, yielding a national percentile, with 99 representing a neighborhood with highest deprivation. The latter was defined based on Rural‐Urban Commuting Area (RUCA) codes, which are defined at the postal code level. Self‐reported race and ethnicity were assessed to inform generalizability, and neither were included in predictive models.

The primary outcome was CGM usage as documented by 20 weeks gestation. This cutoff was selected to allow for patients not presenting to maternal fetal medicine (MFM) care until the second trimester to be fairly assessed, as well as to include those who may have initiated CGM use during the first half of pregnancy, where it potentially may have influenced later second trimester and delivery glycemic management. Primary analysis compared CGM usage during pregnancy by payor status. Secondary analyses compared CGM usage by ADI and rurality. The former was evaluated in a continuous (1–99) and in a binary fashion, with most‐deprived quartile (ADI ≥ 75%) against all others (ADI < 75%). Rurality was likewise compared in a continuous (1–10) and in a binary fashion, with a score of 4 or greater defined as rural. Trends over time were evaluated using delivery year as a continuous variable, and graphs were generated batching deliveries over 2‐year intervals over time (2016–2017, 2018–2019, etc.) for visualization purposes, given relatively small *N* each year. As this was a convenience sample, no a priori power calculation was performed.

Categorical baseline and pregnancy characteristics were compared via chi‐square or Fisher's exact test between commercial and public payor groups, while continuous medians were compared via Wilcoxon rank sum test. The primary outcome was evaluated via regression analysis controlling for maternal age, BMI, and duration of diabetes diagnosis, producing odds ratio (OR) with 95% confidence intervals (CIs). Adoption over time models also included year of delivery. To evaluate the particular relationships between payor status, neighborhood deprivation, rurality, and delivery year, a logistic regression model incorporating payor, ADI, and RUCA code was built, including interaction terms for each along with delivery year to account for changing prevalence over time. Statistical significance was defined at a *p* value < 0.05. Analyses were performed with R version 4.1.3 [33]. The datasets generated and analyzed during this study are available from the corresponding author upon reasonable request.

## 3 RESULTS

**FIGURE 1:**
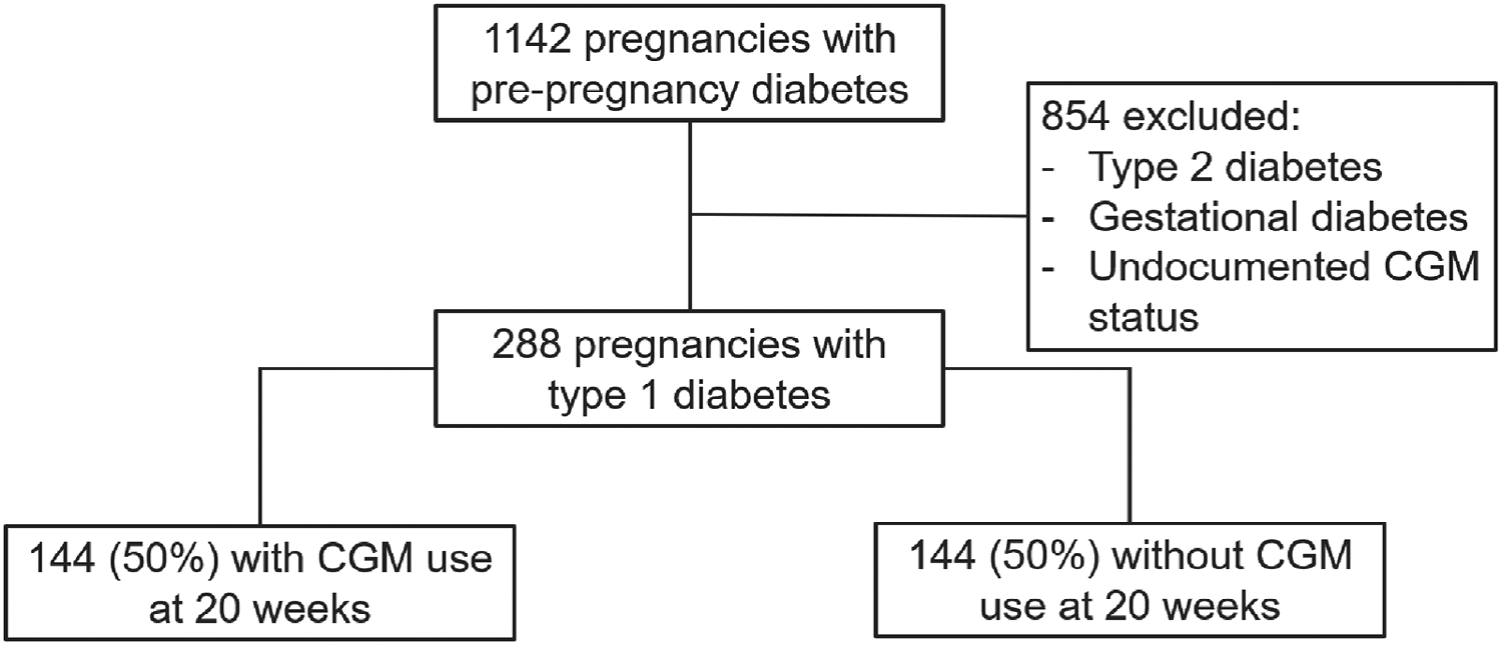
Flow diagram of pregnancies delivering in a large regional health system with associated International Classification of Diseases code associated with type 1 diabetes. CGM, continuous glucose monitor.

Survey of the EMR revealed 1142 deliveries associated with an E10.* ICD‐10 code, implying T1DM. Individual chart review excluded 854 patients who either had been miscategorized as T1DM (predominantly due to single patient encounters where patients with well‐documented type 2 diabetes were mislabeled as having T1DM), delivered prior to 20 weeks gestation, or did not have documentation of CGM status by 20 weeks gestation (primarily due to patients receiving in‐system prenatal care but out of system endocrinology care and whose initial MFM consultation occurred after 20 weeks gestation), resulting in 288 included pregnancies (Figure 1). Patients with public insurance were younger and more likely to be Black (Table 1). Nulliparity, smoking, and chronic hypertension were more common in the public payor group, and people were much more likely to live in high deprivation (45% vs. 8%, *p* < 0.001) and rural neighborhoods (18% vs. 3%, *p* < 0.001). Pump use was more common in the commercial payor group (67.4% vs. 40.3%, *p* < 0.001) and baseline glycemic management was better for those with commercial insurance (HbA1c 6.9% vs. 8.6%, *p* < 0.001).

**TABLE 1:** Characteristics of pregnant patients with type 1 diabetes by private versus public insurance payor.

	Commercial		Public		
	*N* = 144		*N* = 144		*p*
Median age, years (IQR)	31	(27–34)	26	(22–29)	<0.001
Median BMI, kg/m^2^ (IQR)	26.6	(23.6–31.7)	27.5	(23.7–33.3)	0.237
Nulliparous, %	64	44.40%	83	57.60%	0.034
Black, %	24	16.80%	75	52.10%	<0.001
Hispanic, %	1	0.70%	2	1.40%	0.999
Smoker, %	3	2.10%	16	11.10%	0.004
Chronic hypertension, %	21	14.60%	37	25.70%	0.028
Insulin pump, %	97	67.40%	58	40.30%	<0.001
Median duration of diabetes, years (IQR)	13	(9–19)	13	(8–19)	0.363
High neighborhood deprivation (ADI > 75%), %	11	7.60%	65	45.10%	<0.001
Rural, %	4	2.80%	26	18.10%	<0.001
Median baseline hemoglobin A1c % (IQR)	6.90%	(6.3%–8.8%)	8.60%	(7.6%–10.2%)	<0.001

Abbreviations: ADI, Area Deprivation Index; BMI, body mass index; IQR, interquartile range.

**FIGURE 2:**
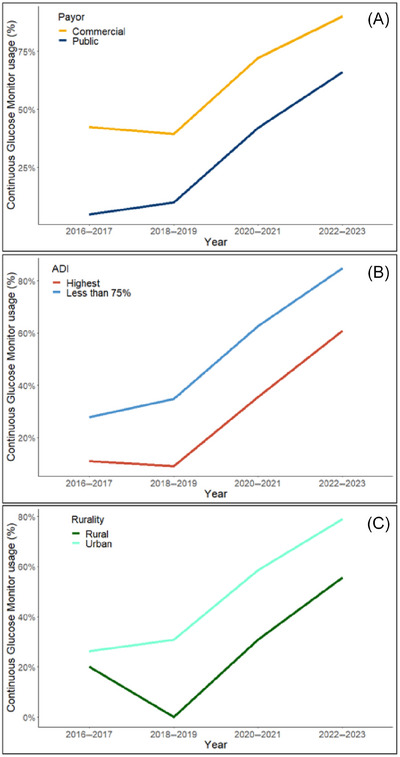
Continuous glucose monitor uptake over time among pregnant patients with type 1 diabetes by individual‐ and neighborhood‐level social determinants of health. (A) Commercial versus public insurance payor status. (B) Home address Area Deprivation Index (ADI) percentile of ≥75% versus <75% (higher percentiles equal higher deprivation). (C) Rural home address versus urban/suburban home address.

When controlling for maternal age, baseline BMI, and duration of diabetes, payor status was associated with lower odds of CGM uptake (39.8% vs. 61.8%, adjusted odds ratio [aOR], 0.39; 95% CI, 0.23–0.66). When adding delivery year into the model, payor status remained associated with decreased CGM use (aOR, 0.25; 95% CI, 0.14–0.47). CGM use increased from 34.6% in 2016 to 75.0% in 2023, associated with a 70% increase in CGM use year over year (aOR, 1.70; 95% CI, 1.44–2.01, Figure 2A). Viewed over time, CGM use increased in each group, though uptake in the public payor group lagged that of the commercial payor group by about 2 years, consistent with Diffusion of Innovations theory (Table 2).

**TABLE 2:** Logistic regression model predicting continuous glucose monitor use by social determinants of health.

Variable	Odds ratio	Lower 95%	Upper 95%
Delivery year	1.832	1.534	2.189
Medicaid payor status	0.274	0.032	2.348
Area Deprivation Index (ADI) percentile	0.947	0.919	0.975
Rural‐Urban Commuting Area (RUCA) code	0.611	0.188	0.198
Medicaid*ADI percentile	1.015	0.983	1.048
Medicaid*RUCA	0.743	0.391	1.411
ADI percentile*RUCA	1.009	0.992	1.026

Next, we evaluated for neighborhood‐level markers of social deprivation. In total, 26.4% (*N* = 76) of included patients lived in high deprivation neighborhoods, defined as ADI > 75%, while 30 (10.4%) lived in rural postal codes. Public payor status was highly associated with high neighborhood deprivation, with 85.5% of those living in high ADI neighborhoods using Medicaid compared to 37.3% in non‐high deprivation neighborhoods (OR, 9.95; 95% CI, 4.95–19.97). Those living in high deprivation neighborhoods used CGM in 38.2% of pregnancies compared to 54.7% in neighborhoods below the highest quartile in ADI (OR, 0.51; 95% CI, 0.30–0.87). Visualized over time, uptake of CGM in the high ADI group again lagged the lower deprivation group by about 2 years (Figure 2B). Public payor status was similarly more common in rural areas (86.7%) compared to urban/suburban zip codes (45.7%; OR, 7.71; 95% CI, 2.62–22.73). Rurality was not significantly associated with CGM use (33.3% vs. 52.3%; OR, 0.46; 95% CI, 0.21–1.01), though with only 30 patients in the rural group, this finding should be interpreted with caution. While not significant, when visualized over time, the trend for CGM uptake among rural patients again lagged that of urban/suburban patients by around 2 years (Figure 2C). In a model including delivery year, payor status, ADI centile, RUCA code, and interaction terms for each, delivery year (aOR, 1.83; 95% CI, 1.53–2.19) and ADI (aOR, 0.94; 95% CI, 0.92–0.97) remained significant predicators of CGM uptake, while rurality (aOR, 0.61; 95% CI, 0.19–1.98) and public payor status (aOR, 0.27; 95% CI, 0.03–2.35, Table 2) did not. The findings demonstrate an 83% year‐over‐year increase in CGM use and indicate that for each increasing ADI centile, odds of CGM use among pregnant people with T1DM decreased by 6%.

## 4 DISCUSSION

Among 288 patients living with T1DM receiving diabetes and pregnancy care at a large regional hospital system, individual‐ and neighborhood‐level social determinants of health were associated with lagging CGM uptake. Those with public payor insurance demonstrated a 61% decreased odds of CGM use compared to commercially insured patients. High neighborhood deprivation was likewise associated with decreased CGM use. While rurality was not significantly correlated with CGM uptake, a low number of rural patients and trend sustain suspicion for a potential association, particularly in light of prior studies of nonpregnant patients [25, 27]. CGM uptake increased substantially through the course of study, from 34.6% in 2016 to 75.0% in 2023. Evaluated by SDOH, both individual and neighborhood social disadvantage appeared to be associated with a 2‐year lag in uptake, consistent with Diffusion of Innovations theory [23].

While associations between SDOH and lagging uptake of medical technology have been characterized, our findings represent the extension of a well‐described phenomenon into a pregnant population. Individual and neighborhood social vulnerability has been associated with poor glycemic management and adverse pregnancy outcomes for those with pregestational diabetes [13, 14]. Tight glycemic management is essential to achieving the best outcomes for both pregnant mothers and their fetuses [10, 12], and CGM use in pregnancy has been associated with both improved glycemic and clinical outcomes [30, 34, 35]. Observed uptake over time demonstrates increasing CGM usage in reproductive‐aged women with T1DM [13], with over 70% of all T1DM pregnancies using CGM in 2022 and 2023. However, inequitable uptake threatens to exacerbate disparities in pregnancy outcomes among those with both individual‐ and neighborhood‐level social deprivation [21].

Understanding particular reasons for lagging uptake by social vulnerability status is essential to achieving more equitable distribution. As LaMOMS has covered CGM devices since the beginning of the study period, the disparity observed among public payor status is not primarily explained by insurance obstacles, raising the question of what other factors may be slowing uptake of CGMs. When individual‐ and neighborhood‐level SDHs were modeled with delivery year, ADI remained a significant predictor of CGM use, implying that neighborhood characteristics may be more important than public insurance status in regard to CGM adoption. Barriers to adoption for individuals’ high social vulnerability may be related to provider bias [22], geographical access to subspecialist care [36], pharmacy‐level factors, and limits in access to the broadband internet that the modern devices depend on for transmitting values to diabetes care teams [37]. Specific interventions to accelerate technology uptake could be focused geographically at either the state or health system level, prioritizing education and access to novel technologies for patients from neighborhoods with high social vulnerability indicators. In regard to an established and well‐disseminated technology like CGM, identifying specific barriers and working toward solutions at the establishment of prenatal (or, optimally, preconception) care should be a priority for providers caring for pregnant patients with T1DM.

This study has important limitations. Retrospective analyses depend upon accurate clinical data entry. Despite individual‐level chart review, CGM status may not be accurately reflected if status was not recorded or if a patient previously on CGM had discontinued use, potentially biasing toward the null hypothesis. Selecting a single timepoint prohibits evaluation of differential timing of initiation through pregnancy. We are unable to speak to specific barriers for included patients and are unable to account for patient preferences regarding the device. While the overall cohort is large and diverse, data are from a single health system, potentially limiting generalizability. Low numbers of rural patients limit power to confidently assess the impact of rurality in our cohort. Finally, we did not evaluate clinical outcomes in this system related to CGM use and cannot speak to the clinical impacts of differences in adoption by SDOH.

The study likewise has strengths. Here we apply the well‐established Diffusion of Innovations theory to evaluate real‐world application of a novel technology associated with improved glycemic outcomes. While the single‐center nature of the study may limit generalizability, even blends in payor status and standardized obstetric guidelines within the system facilitate internal validity. We include only patients with T1DM, avoiding practice variations employed in patients with type 2 and gestational diabetes mellitus. Finally, we identify sociodemographic factors, including payor status and neighborhood deprivation, which can inform specific targets to improve CGM uptake at both the administrative and provider levels.

Individual‐ and neighborhood‐level markers of socioeconomic deprivation are associated with decreased uptake of CGM among pregnant patients with T1DM. While CGM use increased through the study period for each examined group, individual payor status and high neighborhood deprivation were both associated with a 2‐year lag in adoption compared to non‐deprived controls. The observed differences in pregnant patients are consistent with those observed in pediatric and nonpregnant adult patients with T1DM and represent a potential target for reducing disparities in glycemic management and adverse pregnancy outcomes.

## AUTHOR CONTRIBUTIONS

Frank B. Will Williams, Kali Juracek, John A. Morgan, James D. Toppin, Joseph R. Biggio, and Shannon M. McCloskey were involved in the conception, design, and conduct of the study and the analysis/interpretation of the results. Frank B. Will Williams wrote the first draft of the manuscript, and all authors reviewed, edited, and approved the final version of the manuscript. Frank B. Will Williams is the guarantor of this work and, as such, has full access to all the data in the study and takes responsibility for the integrity of the data and the accuracy of the analysis.

## CONFLICT OF INTEREST STATEMENT

The authors declare no conflicts of interest.

## Data Availability

The data that support the findings of this study are available from the corresponding author upon reasonable request.
